# Investigation of the diversity, equity, inclusion, and belonging within the undergraduate student population within the Department of Animal Science at Iowa State University

**DOI:** 10.1093/tas/txae100

**Published:** 2024-06-26

**Authors:** Kelsi A Carlson, Jennifer M Bundy, Michael J Martin, Scott W Smalley, Anna K Johnson

**Affiliations:** Department of Agricultural Education and Studies, College of Agriculture and Life Sciences, Iowa State University, Ames, IA 50011, USA; Department of Animal Science, College of Agriculture and Life Sciences, Iowa State University, Ames, IA 50011, USA; Department of Agricultural Education and Studies, College of Agriculture and Life Sciences, Iowa State University, Ames, IA 50011, USA; Department of Agricultural Education and Studies, College of Agriculture and Life Sciences, Iowa State University, Ames, IA 50011, USA; Department of Animal Science, College of Agriculture and Life Sciences, Iowa State University, Ames, IA 50011, USA

**Keywords:** acceptance, comfort, opportunities, success, tools

## Abstract

Iowa State University (ISU) provides resources for diversity, equity, inclusion, and belonging (DEI-B) to provide students with a comfortable academic home regardless of their demographics or prior experiences. The objective of this study was to investigate undergraduate students’ DEI-B perspectives in the Department of Animal Science at ISU. One survey instrument was developed containing 14 questions that covered demographics, feelings of inclusion, comfort-seeking tools, and ways to improve DEI-B. Answer choices were either multiple choice, 1 to 5 sliding scale, or a specified text sliding scale. Eligible participants were undergraduate students enrolled in Animal or Dairy Science (*n* = 974). Demographics and comfort-seeking tools will be presented descriptively. Inclusion at the start and after 2 yr were compared using six different linear models. A variable was deemed significant if the *P*-value was ≤ 0.05. A total of 383 students (88% of total respondents) completed 50% or more of the inclusion questions. Seniors had the highest response rate. More students reported coming from a rural background. Primary species of interest was companion animals. There were no observed differences in feelings of inclusion in classes, with peers, or with faculty for hometown, admission type, ethnic group, and first generation when students started (*P *≥ 0.067). There was a difference for primary species of interest (*P* ≤ 0.011) and with female students feeling less included (*P* ≤ 0.039). There were no observed differences after 2 yr in classes, with peers, or with faculty for classification year, admission type, or first generation (*P* ≥ 0.088). Suburban students felt the least included in classes compared to rural and urban students (*P* ≤ 0.036). Female students felt less included in all three categories (*P* ≤ 0.017). The majority of students reported having companion animal experience but almost half reported having no experience with livestock prior to ISU. A total of 51% of students said they never considered transferring to another major and 48% indicated that they plan to pursue a career in veterinary medicine. A total of 75% of students felt inclusion could be improved by creating more hands-on opportunities and 60% suggested the department provide more study space. In conclusion, the Department of Animal Science at ISU has some effective inclusion practices but needs to evolve and improve in its DEI-B practices for the undergraduate student population.

## Teaser

Animal Science undergraduate students are the future leaders in agriculture. Helping them succeed and find a place when they may not be a “traditional” student is critically important and is foundational for DEI-B efforts.

## Introduction

Iowa State University (ISU) provides resources for diversity, equity, inclusion, and belonging (DEI-B) to provide students a comfortable academic home in which they can succeed regardless of their demographics or previous experiences. The University’s DEI-B statement is “Iowa State University defines diversity broadly to include ability, age, gender, culture, race, religion, sexual orientation, and socio-economic background. Equity refers to fairness and social justice, such as treating people fairly while recognizing different people’s needs may differ significantly. Inclusion means not only welcoming and including but creating and ensuring conditions for full and meaningful participation so that all people feel they are welcome and valued as members of the wider University community. To create an environment that values and promotes diversity, equity, and inclusion (DEI) requires action and engagement” (ISU, 2024a).

Every college at ISU has a variety of DEI-B resources and departments select how much to implement or expand. The Animal Science Department’s DEI-B vision statement is *“*…to be globally recognized as an entity where diversity of background, identity, ideas, and expertise is encouraged, pursued and valued,” and the mission statement is to “Empower and utilize our diverse strengths and identities to achieve global excellence in the teaching, research and outreach/extension missions of the department, college and university” (ISU, 2024b).

The Department of Animal Science has undergraduate students who come from diverse demographics. The tenth day registrar undergraduate Animal Science list from Spring 2022 reported a total of 974 undergraduate students. Within this population, there were 159 freshmen, 210 sophomores, 257 juniors, and 348 seniors. A total of 801 students were of white ethnicity, 170 were of either Asian, black, Hispanic, or two+ races, and three did not display their ethnicities. Such demographics have brought forth questions related to the departments DEI-B efforts and what opportunities are being provided to undergraduates for them to succeed. Therefore, the objective of this study was to investigate undergraduate students’ DEI-B perspectives in the Department of Animal Science at ISU.

## Materials and Methods

This study was reviewed and approved as exempted research by the ISU Institutional Review Board (IRB: 22-002) for Human Subjects Research and complied with CFR 45 Part 46.

### Qualtrics Survey Building

One survey was created using Qualtrics software (Version February 2022; Provo, UT, USA). A total of 14 questions were displayed if applicable to undergraduate students based on their selection of academic rank. Demographic questions included academic rank, hometown description, academic/major clubs, primary species of interest, previous livestock, and companion animal experience, and career goals. Four inclusion questions were included at the start and more than 2 yr in the department and rating how students were being treated in and outside of a class by faculty and peers. One comfort question asked how they felt about asking questions to faculty in and outside of class. One question addressed what the department can provide to help with inclusiveness and one question asked students if they had considered changing majors. Answer choices were either multiple choice, 1 to 5 sliding scale, or a specified text sliding scale (Table 1).

**Table 1.** Undergraduate student survey questions, Department of Animal Science at ISU

No.	Question	Options	Scale
1	What is your academic rank?	a. Freshman b. Sophomore c. Junior d. Senior	NA
2	How would you describe your hometown?	a. Rural b. Suburban c. Urban	NA
3a^1^	What extra-curricular activities are you currently involved in?	Select all that apply:	NA
		a. Greek life b. Academic/major-related club# c. Social club d. Volunteering e. Athletics/intramural f. Religious group g. Other (text box)	
3b	What academic/major-related club are you in?	Select all that apply:	NA
		a. Block and Bridle b. Dairy Science c. Pre-Veterinary Club d. Veterinarians without Borders	
4	What is your primary species of interest in Animal Science?	a. Beef b. Companion (not horses) c. Dairy d. Horses e. Laboratory animals f. Poultry g. Small ruminants (sheep/goats) h. Swine i. Other (text box)	N/A
5	Did you have any previous livestock experience (this excludes horses) before entering the Department of Animal Science?	Select all that apply:	N/A
		a. Yes, at home b. Yes, from a job c. No, none until the Animal Science Department	
6	Did you have any previous companion animal experience (this includes horses) before entering the Department of Animal Science?	Select all that apply:	N/A
		a. Yes, at home b. Yes, from a job c. No, none until the Animal Science Department	
7	Rate your level of inclusion as to how you felt in the following areas when starting in the Department of Animal Science.	a. Topics and discussions in animal science classes b. With peers in animal science-related activities c. Faculty (this includes professors, advisors, and teaching)	1 to 5^4^
8^2^	Rate your level of inclusion of how you felt in those areas after being in the Department of Animal Science for more than 2 yr.	a. Topics and discussions in animal science classes b. With peers in animal science-related activities c. Faculty (this includes professors, advisors, and teaching)	1 to 5^4^
9	Rate how you feel students in the department are being treated:	a. In classes by faculty (this includes professors and teaching assistants) b. Outside of class by faculty (this includes professors, advisors, and assistants)	1 to 5^5^
10	Rate how you feel students in the department are being treated in:	a. In classes by peers b. Outside of class by peers (in clubs, social activities, etc.)	1 to 5^6^
11	Have you ever considered transferring to another major?	a. All of the time b. I consider it frequently c. Sometimes d. It has come to my mind once or twice e. Never	N/A
12	Rate your comfort level when asking questions in:	a. In class to faculty (this includes professors and teaching assistants) b. Outside of class to faculty (this includes advisors, professors, and teaching assistants)	1 to 5^7^
13	What are your career goals after completing your education?	a. Veterinary school b. Graduate school c. Into the industry	N/A
14	Which of the following can the department provide to help with inclusiveness?	Select all that apply:	N/A
		a. Provide spaces where students can relax and/or work together b. Include artifacts around the department that shows Animal Scientists whom have come from diverse backgrounds c. Create a peer mentoring program beyond the ﬁrst semester where students with previous experience can meet with those of less experience d. Create opportunities for students to work on university farms to garner hands-on livestock experience e. Other (text box)	

^1^If academic/major-related club was selected, participants were prompted to question 3b.

^2^Asked to those that selected academic rank of junior or senior on question 1.

^3^Asked to those that selected academic rank of freshman, sophomore, junior, or senior on question 1.

^4^Sliding scale as follows: 1 = not included at all; 2 = not usually included; 3 = moderately included; 4 = usually included; 5 = very included.

^5^Sliding scale as follows: 1 = all students are treated the same with no modiﬁcation based on their previous animal science experience; 2 = most students are treated the same regardless of their previous animal science experience; 3 = This has not been an observed issue; 4 = some students receive tailored assistance accordingly to their previous animal science experience; 5 = every student gets tailored assistance based on their speciﬁc previous animal science experience.

^6^Sliding scale as follows: 1 = no students are accepted by peers; 2 = some students are accepted by peers; 3 = this has not been an observed issue; 4 = most students are accepted and valued by peers; 5 = all students are accepted and valued by peers.

^7^Sliding scale as follows: 1 = very uncomfortable; 2 = slightly uncomfortable; 3 = moderately comfortable; 4 = mostly comfortable; 5 = very comfortable.

### Participants

Eligible participants were undergraduate students enrolled in either the Animal Science or Dairy Science major regardless of ability, age, gender, culture, race, religion, sexual orientation, and socioeconomic background (*n* = 974).

### Survey Distribution and Completion

An email list serve was created from the tenth day undergraduate enrollment list, spring 2022. Undergraduate students (*n* = 974) were contacted by the undergraduate coordinator via email with the survey link, on February 4, 2022. A reminder email was sent at one and 2 wk, respectively, after the initial email to non-responders. The survey was closed on February 23, 2022.

Participants read and agreed to a consent statement prior to starting the survey. To ensure anonymity, no identifying information was collected by the Qualtrics platform.

### Statistical Analysis

Student demographics, participation in extra-curricular activities, academic/major-related clubs, and primary species of interest will be presented descriptively (numbers and percentages). Previous livestock and companion animal experience before entering the Department of Animal Science considered transferring to another major, and career goals after completing the animal science education will be presented descriptively (number and percentages). Students ranked their perceived level of inclusion in topics and discussions in class, among peers in animal science-related activities, and with faculty. All respondents provided these rankings for the start of their time in the major. Juniors and seniors also provided these rankings after 2 yr of experience in the major. To determine significance of various demographic factors, six different linear models were created using inclusion rank as the dependent variable. These six different dependent variables were inclusion level when starting in classes, starting with peers, starting with faculty, and, after two or more years in classes, with peers, and with faculty, respectively. The fixed effects that were fit into the model included primary species of interest (beef, companion, dairy, horses, laboratory animals, poultry, small ruminants, swine, other), sex (male and female), hometown (rural, suburban, urban), academic rank (freshman, sophomore, junior, senior), ethnicity (Asian, Black, Hispanic, prefer not to answer, two + races, White), first generation status, and admission type (direct from high school or transfer student). Based on these models, significance of fixed effect, least squares means, and standard errors were calculated. A variable was deemed significant if the *P*-value was ≤ 0.05.

## Results

A total of 436 students opened the survey with 383 (**88%)** students completing 50% or more of the inclusion questions. **This resulted in 383 student responses included in the analysis.** Answers from all student classifications were received, with seniors having the highest response rate and freshman the lowest. More students identified as coming from a rural background with the least identifying from an urban background. Students selected a range of extra-curricular activities that they participated in but the majority identified with academic/major-related clubs. When asked about their primary species of interest, a higher response rate was selected for companion animals followed by beef. Horse, swine, and dairy were fairly evenly selected at ~10% each (Table 2).

**Table 2.** Demographic undergraduate student responses (number and percentage) within the Department of Animal Science at Iowa State

	Responses (*n*)	Total population (%)^1^
Academic rank		
Freshman	74	19
Sophomore	94	25
Junior	103	27
Senior	112	29
Hometown		
Rural	217	57
Suburban	114	30
Urban	50	13
Did not answer^2^	2	1
Extra-curricular		
Greek life	43	11
Academic/major-related club	241	63
Social club	81	21
Volunteering	106	28
Athletics/intramural	65	17
Religious group	67	17
Other	44	11
Did not answer^2^	50	13
Primary species of interest		
Beef	72	19
Companion	129	34
Dairy	35	9
Horses	46	12
Laboratory animals	6	2
Other	22	6
Poultry	10	3
Small ruminants	24	6
Swine	39	10

^1^Total population is defined as the percentage that each group represents out of the total number of respondents (*n* = 383).

^2^Students were able to continue with the survey if they did not want to answer these questions. “Did not answer” represents those students who chose to move on without answering.

There were no observed differences in feelings of inclusion when starting within the department for hometown, admission type, ethnic group, and first generation (*P* ≥ 0.067). Primary species of interest had a significant effect on feelings of inclusiveness in classes, with peers, and with faculty (*P* ≤ 0.011). Students who identified their primary species of interest as beef indicated more inclusiveness overall categories. Students who identified with companion and laboratory animals reported lower levels of inclusion compared with the other species of interest when in classes and among peers. However, those identifying with laboratory animals reported the highest level of inclusion with faculty. There was a difference for classification year for peers and faculty with seniors feeling less included (*P* ≤ 0.044). There was a difference with male and female students in classes, with peers, and with faculty, with females feeling less included (*P* ≤ 0.039; Table 3).

**Table 3.** LSMeans and SE for undergraduate Animal Science students at ISU when asked “Rate your level of inclusion as to how you felt in topics and discussions in animal science classes, with peers in animal science related activities, and with faculty when starting in the Department of Animal Science” in Qualtrics using a Likert scale^1^

		Classes		Peers		Faculty	
Measure	No.	LSMeans SE	*P* value	LSMeans SE	*P* value	LSMeans SE	*P* value
Hometown^2^							
Rural	217	3.95 ± 0.21		3.74 ± 0.22	0.8077	4.17 ± 0.23	0.1875
Suburban	114	3.67 ± 0.21	0.0672	3.68 ± 0.22		3.98 ± 0.23	
Urban	50	4.03 ± 0.23		3.57 ± 0.24		4.24 ± 0.25	
Did not answer	2	3.89 ± 0.71		3.56 ± 0.74		3.08 ± 0.77	
Primary species of interest							
Beef	72	4.20 ± 0.26		4.02 ± 0.27		4.02 ± 0.28	
Companion^3^	129	3.60 ± 0.23		3.43 ± 0.25		3.63 ± 0.26	
Dairy	35	3.74 ± 0.27		3.66 ± 0.29		3.78 ± 0.30	
Horses	46	3.74 ± 0.27		3.68 ± 0.29		3.45 ± 0.30	
Laboratory animals	6	3.68 ± 0.44	0.0011	3.22 ± 0.47	0.0078	4.06 ± 0.48	0.0110
Primary species of interest							
Other	22	3.50 ± 0.29		3.39 ± 0.31		3.56 ± 0.32	
Poultry	10	4.33 ± 0.37		3.47 ± 0.39		4.10 ± 0.41	
Small ruminants	24	4.04 ± 0.30		3.93 ± 0.32		4.00 ± 0.33	
Swine	39	4.11 ± 0.27		3.94 ± 0.29		4.23 ± 0.30	
Classification year							
Freshman	74	4.09 ± 0.27	0.0659	3.82 ± 0.28	0.0045	4.07 ± 0.29	0.0435
Sophomore	94	3.72 ± 0.25		3.73 ± 0.27		3.75 ± 0.28	
Junior	103	3.94 ± 0.25		3.66 ± 0.26		3.97 ± 0.27	
Senior	112	3.78 ± 0.24		3.34 ± 0.25		3.69 ± 0.26	
Sex							
Female	336	3.73 ± 0.23	0.0392	3.48 ± 0.25	0.0394	3.68 ± 0.26	0.0394
Male	47	4.04 ± 0.26		3.80 ± 0.27		4.06 ± 0.28	
Admission type							
High school	326	3.86 ± 0.23	0.7811	3.65 ± 0.24	0.8618	3.86 ± 0.25	0.8618
Transfer	57	3.90 ± 0.26		3.62 ± 0.27		3.88 ± 0.28	
Ethnic group							
Asian	2	3.76 ± 0.69		3.59 ± 0.73		4.00 ± 0.76	
Black	4	3.86 ± 0.48		3.31 ± 0.50		4.59 ± 0.52	
Hispanic	30	3.44 ± 0.26	0.0803	3.23 ± 0.27	0.2007	3.32 ± 0.28	0.2007
Prefer not to answer	4	4.57 ± 0.51		4.11 ± 0.54		4.20 ± 0.56	
Two+ races	11	3.69 ± 0.31		3.89 ± 0.33		3.37 ± 0.34	
White	332	3.97 ± 0.21		3.70 ± 0.22		3.74 ± 0.23	
First generation							
No	299	3.91 ± 0.24	0.6974	3.66 ± 0.25	0.7250	3.88 ± 0.26	0.7250
Yes	84	3.86 ± 0.25		3.61 ± 0.26		3.86 ± 0.27	

^1^Undergrduate Animal Science students were asked to rank questions on a Likert scale consisting of 1 = strongly disagree; 2 = disagree; 3 = neither agree or disagree; 4 = agree; 5 = strongly agree. Students were able to continue with the survey if they selected “Did not answer.”.

^2^Hometown was defined and interpreted by the students.

^3^Companion animals does not include horses.

Classification year, admission type, or first generation did not have effects on feelings of inclusion once a student had been within their major for more than 2 yr (*P* ≥ 0.088). With hometown there was no difference in inclusiveness with peers and faculty (*P* ≥ 0.441), but hometown was a significant effect on feelings of inclusiveness within classes with suburban students feeling the least included compared to rural and urban students (*P* ≤ 0.036). There was no difference for primary species of interest in classes (*P* = 0.07), but students did report differences in inclusion when considering peers and faculty (*P* ≤ 0.038). Students who identified horses and dairy cattle as their primary species of interest felt the most included with peers (*P* = 0.028). Students who identified laboratory animals as their primary species of interest reported high inclusion with faculty while students identifying companion animals being less included by faculty (*P* = 0.038). There was a difference between male and female students overall three categories, with females feeling less included (*P* ≤ 0.017). Although no differences were reported for ethnic group, numerically Hispanic students reported the lowest level of inclusion with peers (2.95; *P* ≤ 0.076). There was a difference between white students and students from minoritized groups in feelings of inclusiveness when interacting with faculty (*P* = 0.026). Students identifying as Hispanic and two + races reported the lowest level of inclusion, and black students reported the highest level of inclusion (Table 4).

**Table 4.** LSMeans and SE for undergraduate Animal Science students at ISU when asked “Rate your level of inclusion as to how you felt in topics and discussions in animal science classes, with peers in animal science related activities, and with faculty after being in the Department of Animal Science for more than two years” in Qualtrics using a Likert scale^1^

		Classes		Peers		Faculty	
Measure	No.	LSMeans SE	*P* value	LSMeans SE	*P* value	LSMeans SE	*P* value
Hometown^2^							
Rural	122	3.80 ± 0.27	0.0356	3.51 ± 0.35	0.6287	4.19 ± 0.32	0.4410
Suburban	69	3.58 ± 0.27		3.39 ± 0.35		3.95 ± 0.32	
Urban	19	3.80 ± 0.32		3.26 ± 0.41		4.19 ± 0.37	
Did not answer^6^	2	5.36 ± 0.62		4.09 ± 0.79		4.08 ± 0.72	
Primary species of interest							
Beef	43	4.29 ± 0.26		3.86 ± 0.33		4.18 ± 0.30	
Companion^3^	68	3.96 ± 0.23		3.49 ± 0.29		3.83 ± 0.26	
Dairy	22	4.47 ± 0.26	0.0721	3.98 ± 0.33	0.0272	4.40 ± 0.30	0.0377
Horses	28	4.09 ± 0.27		3.99 ± 0.35		4.07 ± 0.31	
Classification year							
Laboratory animals	5	3.91 ± 0.41		3.42 ± 0.52		4.50 ± 0.47	
Other	7	3.60 ± 0.37		2.77 ± 0.47		3.30 ± 0.43	
Small rum.	14	4.09 ± 0.31		3.65 ± 0.39		3.94 ± 0.36	
Swine	21	4.30 ± 0.28		3.90 ± 0.36		4.20 ± 0.33	
Sex							
Sophomore	3	3.57 ± 0.51		2.64 ± 0.65		3.37 ± 0.59	
Junior	71	4.44 ± 0.23	0.2613	4.09 ± 0.30	0.0880	4.52 ± 0.27	0.1412
Senior	138	4.39 ± 0.23		3.96 ± 0.29		4.40 ± 0.27	
Female	186	3.83 ± 0.25	0.0009	3.29 ± 0.32	0.0172	3.82 ± 0.29	0.0069
Male	26	4.43 ± 0.25		3.84 ± 0.32		4.38 ± 0.29	
Admission type							
High school	165	4.21 ± 0.23	0.2566	3.60 ± 0.30	0.6219	4.19 ± 0.27	0.2225
Transfer	47	4.06 ± 0.25		3.52 ± 0.32		4.01 ± 0.29	
Ethnic group							
Asian	2	4.72 ± 0.59	0.0052	4.09 ± 0.75	0.0755	4.30 ± 0.68	0.0261
Black	3	4.10 ± 0.46		3.78 ± 0.59		5.29 ± 0.53	
Hispanic	19	3.84 ± 0.27		2.95 ± 0.34		3.41 ± 0.31	
Prefer not to answer	3	4.21 ± 0.50		3.68 ± 0.64		3.76 ± 0.58	
Two+ races	3	3.39 ± 0.50		3.13 ± 0.64		3.84 ± 0.58	
White	182	4.55 ± 0.22		3.73 ± 0.28		4.00 ± 0.26	
First generation							
No	160	4.12 ± 0.24	0.8022	3.55 ± 0.30	0.8963	4.12 ± 0.27	0.8166
Yes	52	4.15 ± 0.25		3.57 ± 0.32		4.08 ± 0.29	

^1^Undergraduate Animal Science students were asked to rank questions on a Likert scale consisting of: 1 = strongly disagree; 2 = disagree; 3 = neither agree or disagree; 4 = agree; 5 = strongly agree.

^2^Hometown was defined and interpreted by the students.

^3^Companion animals does not include horses.

^4^Students were able to continue with the survey if they did not want to answer these questions. “*Did not answer*” represents those students who chose to move on without answering.

**Figure 1.**
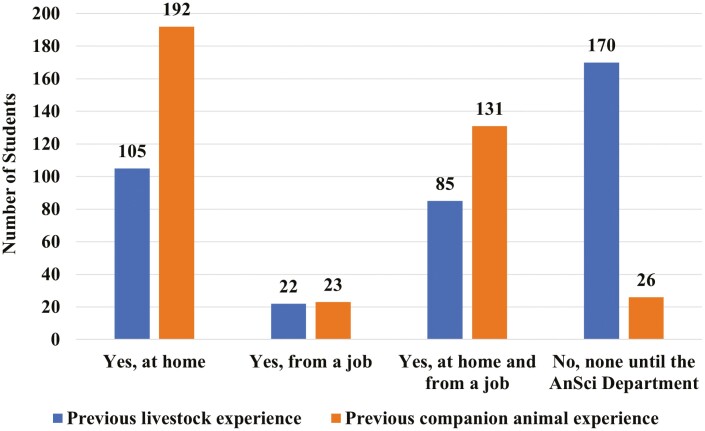
Number of undergraduate Animal Science students at ISU when asked “Did you have any previous livestock experience (this excludes horse) before entering the Department of Animal Science” (*n* = 382), and “Did you have any previous companion animal experience (including horses) before entering the Department of Animal Science?” (*n* = 372)

The majority of students had a strong comfort level when asking questions in (n = 154) and outside (n = 237) of class to faculty. The majority of students reported having companion animal experience from home followed by a combination of home and job(s). Almost half of students reported having no experience with livestock until they got to the Animal Science department (Fig. 1).

**Figure 2.**
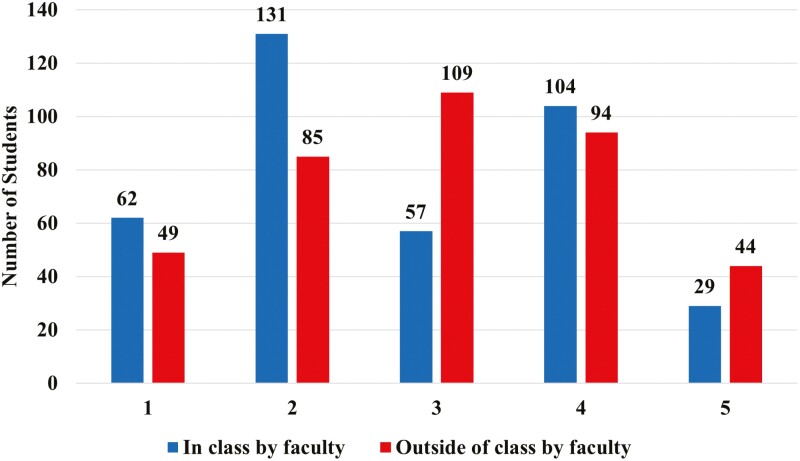
Number of undergraduate Animal Science students at ISU when asked “Rate how you feel students in the department are being treated in these areas.” (*n* = 383,381)

When asked about equity in class by faculty, 50% of students identified with “treated the same with no modification based on their previous animal science experience” and “treated the same regardless of their previous animal science experience.” Inclusion by faculty outside of class was 35% for “treated the same with no modification based on their pervious animal science experience” and “treated the same regardless of their previous animal science experience” When asked about equity in class by faculty, 35% of students identified with “some students receive tailored assistance according to their pervious animal science experience” and “every student gets tailored assistance based on their specific animal science experiences.” Inclusion of faculty outside of class, 36% of students identified with “some students receive tailored assistance according to their pervious animal science experience” and “every student gets tailored assistance based on their specific animal science experiences” (Fig. 2).

**Figure 3.**
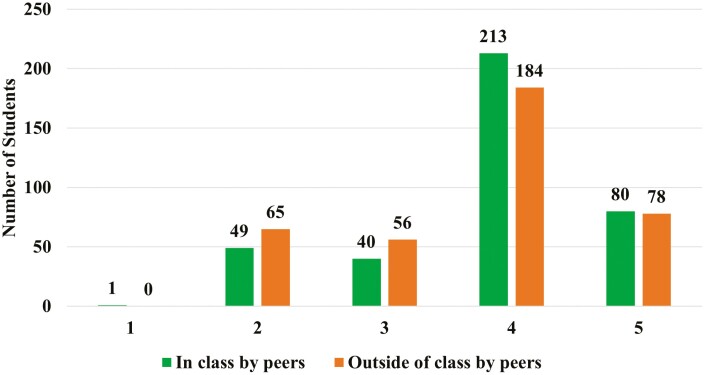
Number of undergraduate Animal Science students at ISU when asked “Rate how you feel students in the department are being treated in these areas.” (*n* = 383)

When asked about inclusion in class by peers 77% responded that “most students are accepted and valued by peers” and “all students are accepted and valued by peers.” A total of 88% responded that “most students are accepted and valued by peers” and “all students are accepted and valued by peers” outside of class. Fewer students reported “some students are accepted by peers in class”, and 12% and 17% (Fig. 3), respectively.

**Figure 4.**
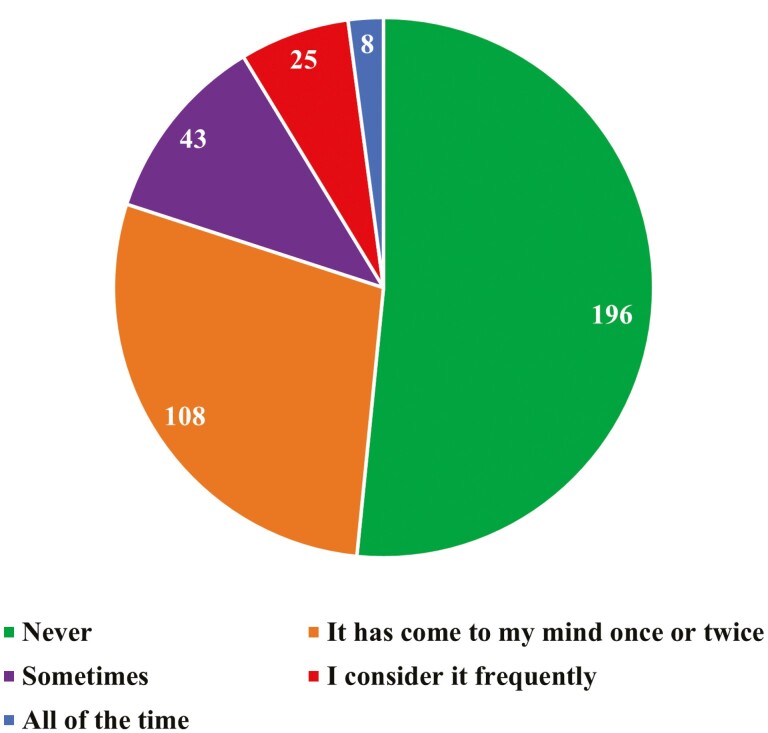
Number of undergraduate Animal Science students at ISU when asked “Have you ever considered transferring to another major?” (*n* = 383)

**Figure 5.**
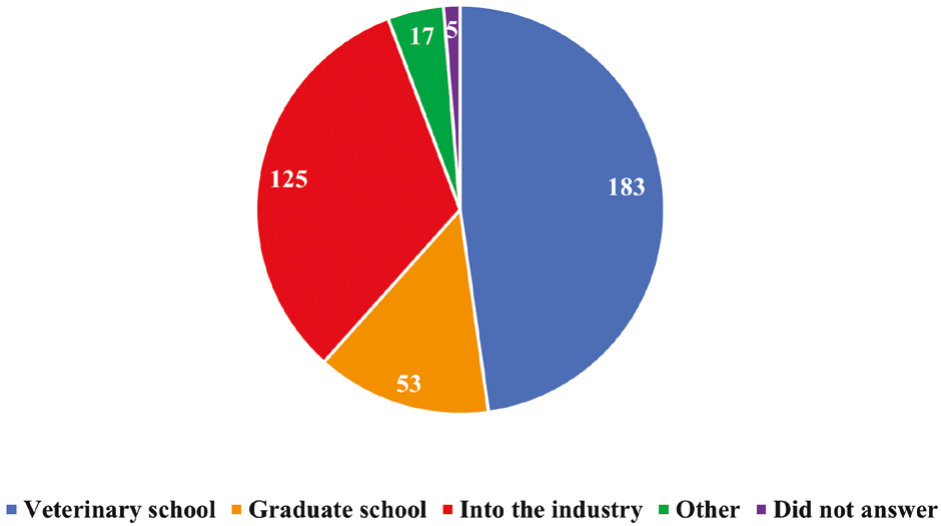
Number of undergraduate Animal Science students at ISU when asked “What are your career goals after completing your education?” (*n* = 383)

**Figure 6.**
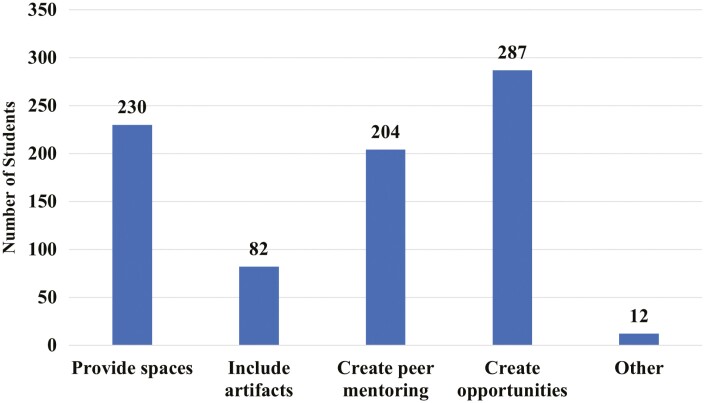
Number of undergraduate Animal Science students at ISU when asked “Which of the following can the department provide to help with inclusiveness?” (*n* = 383)

A total of 51% of students said they had never considered transferring to another major, followed by 28% saying it had come to their mind once or twice (Fig. 4). A total of 48% of students indicate that post-graduation they wish to pursue a profession in veterinary medicine, followed by industry positions (33%) and graduate school (14%; Fig. 5). When students were asked to “select all that apply” as it related to what the department could provide to help with inclusiveness, 75% selected to create more hands-on opportunities, followed by 60% selecting provide spaces (Fig. 6).

## Discussion

The objective of this study was to investigate undergraduate students DEI-B perspectives in the Department of Animal Science at ISU. Survey research can be challenging due to low response rates (5% to 30%). However, in this study ~45% of the undergraduates started and, 88% of students completed 50% or more of the survey. These high completion levels could be interpreted as DEI-B being a subject matter of importance to them.

When reflecting on how undergraduates self-identified their hometown, students did not align with Iowa and the national demographics. Almost 60% of undergraduates identified as rural with just over 40% identifying as urban and suburban. Although Iowa is an agricultural state, general populace has been shifting from rural to urban/suburban. In 2020, Iowa’s urban population was 2,014,831 (63.2%) with the rural portion at 1,175,538 (36.8%; Burke, 2020). Multiple push factors have driven this migration from rural to urban settings and these factors may apply to the animal science undergraduate population. Goetz and Debertin (1996) and Barkley (1990) identified relatively higher unemployment in rural areas and better job opportunities in urban areas. Limited educational opportunities (Huang et al., 2002), educational attainment, home ownership, amenities access (i.e., health care), entertainment options, and labor market characteristics also play a significant role in rural outmigration (Marre, 2009). It would be wise for animal science departments to monitor how undergraduates self-identify their hometown and to ascertain their exposure to livestock and animal agriculture in general when entering into the degree.

In this work ~45% of undergraduates entering into the animal science degree selected “no livestock experience until entering the department” and the vast majority of students noted companion animals as their primary species of interest. With less livestock exposure, these students may feel at a disadvantage to their peers, but faculty can work to rectify this misconception and the department can create more hands-on livestock opportunities. One example that the Department of Animal Science at ISU invested in was creating a Freshman level course that provides students the opportunity to gain extensive livestock handling experience (McNeil et al. 2015). Bundy et al., (2019) reported that upon course completion, students reported that their comfort level while handling livestock had increased for all livestock species. The largest increases were observed with poultry (37.8% to 66.9%) and dairy cattle (49.3% to 84.3%). Of the 75 students polled, 96% felt that the hands-on approach was beneficial at reinforcing lecture material, and 100% reported that they were more likely to voluntarily interact with livestock inside or outside of the classroom setting after course completion.

When considering the primary species of interest, this survey forced a student to select one animal. Although companion animal was the most selected, a student may also have an interest in other species. Future work asking students to rank all species by interest would be useful. At ISU, the Department of Animal Science has been considering how to balance livestock and companion animal opportunities for students to tailor their educational pursuits and to make them feel included. Faculty have been hired to teach companion animal courses and the department has created five certificates that allow students to focus their coursework on a specific industry (Auwerda et al., 2021; Bobeck et al., 2021; Bundy et al., 2021a, 2021b; Burgett et al. 2022). This gives students the credentials to document their specialization and makes them more marketable within those industries. Another option to support students who have an interest in companion animals is the ACE dog agility program. Since 2022, 198 undergraduates, 8 graduate students, and 23 shelter foster dogs have been enrolled in this program. Furthermore, students have worked closely with 14 retrieving freedom dogs (Retrieving Freedom, 2024). These programs have provided students with expertise in outreach, agility, and obedience classes. Since the inception of this program, five classes are now offered.

Another factor that may affect how included a student feels is how they began their undergraduate career. Currently, undergraduates can join the department right after high school or they can transfer in after a 2-yr community college experience. The transfer has been identified as “transfer shock”, which is defined as “a temporary dip in grades during the first semester or two, along with some social disorientation” (Nesbit, 2019). Transfer shock is usually focused around social, psychological, academics, and environment (Laanan, 2007). Encouragingly, all undergraduates in this survey regardless of entering the department directly from high school or transferring in, did not indicate differences in inclusion. The department has made concerted efforts to improve the experiences of incoming freshmen and transfers students (Bundy, 2018; Siberski and Bundy, 2019). A robust orientation was created in 2018 that focuses solely on transfer students and common transfer student issues. The Animal and Dairy Science learning community is a required component of both the freshmen and transfer orientation courses. The learning community uses peer mentors to introduce resources, common struggles, and informal advisement. Incoming students are matched to peer mentors who have similar interests as them. Students are required to participate in peer mentoring groups of 8 to 12 students once a week as part of the orientation course (Bundy, 2018).

When students were asked how they were being treated by faculty, this considered the concept of equity. Equity has been defined as “Commitment to fostering a climate where all individuals have access and opportunity to fully participate in the educational and working environment” (ISU, 2023). Animal science class sizes vary from 25 to 300 so tailored assistance is not always available. However, a third of students reported in class, tailored assistance is given when needed for students to succeed. Additionally, 36% of the students recognized that specific tailored assistance is available outside of class. Students indicated inclusion in and out of class with peers which could be influenced by study groups that transfer relationships from in class to out of class, classmates that are involved in the same clubs, and peer mentoring groups. When students were asked if they had considered transferring out of the department, 80% of students had never or rarely considered it. However, for students who answered “sometimes”, “consider it frequently”, or “all of the time”, 31 out of 83 students (37%) identified companion animals as their primary species of interest.

When considering ethnic group identification and male versus female, two interesting results were collected. Our findings indicated that students identifying as Hispanic and female overall felt less included in classes, by peers, and with faculty at the start and after 2-yr within the department. In spring of 2022 the undergraduate population of the department is 10.4% Hispanic but 81% female. Bundy et al., (2019) reported that female students in the department had more perceived academic struggles than males, despite earning a higher GPA than males. Therefore, these academic challenges may partially explain these results. However, other considerations as to why female undergraduates feel excluded may be that females interested in animal science are not offered the same opportunities to work on livestock farming operations or that females are not aware of their peers holding agricultural leadership roles and cannot visualize a career in this profession. These findings agree with Guterl (2014) who noted that the community of scientists has never been as diverse as the population at large in the U.S. Furthermore, Reese (2020) noted that the current culture and practices of science are not always welcoming, leaving women, people of color, individuals with disabilities, and those who identify as LGBTQIA + minimized or left out completely. Future focal work to dive deeper into why Hispanic students and females feel less included in animal science is warranted.

When asking students what the department could consider to improve DEI-B efforts, students ranked create more study space and collaboration and allow the peer mentor program to extend b**e**yond the first semester. Pierce et al. (submitted for publication) probed deeper into these DEI-B challenges with the same undergraduate population used in this work. Interestingly the farm versus non-farm background was noted as an inclusion issue by 20% of the students, 9% noted that instructors unfairly made assumptions about a student’s livestock background and 20% wanted additional companion animal options to be added to the curriculum. A total of 11% asked for more diverse representation in speakers, artwork, and artifacts around the department. Items related to work space, peer mentoring support, and academic clubs not being welcoming combined at 13%. The department has been making changes based on this feedback. Screens that display hands-on animal opportunities for undergraduate students are placed around the department. Internship, scholarship, and job opportunities are published once in every 2 wk undergraduate newsletter and on all departmental social media pages. To extend opportunities for peer mentoring and provide accessible academic support, the department has opened an Animal Science student help room fall 2023. This help room offers free drop-in tutoring and a space to host office hours for peer mentors and teaching assistants. To create a culture within the department that focuses on equal and equitable opportunities for students who are from agriculture farm backgrounds and those without prior experience, the department also plans to implement a research matching program. The program will allow students to apply for research experience, regardless of the Animal Science discipline. Advising staff members will then match the students’ interest to faculty conducting research in that area and notify the faculty of the student’s interest.

In conclusion, the Department of Animal Science at ISU has some effective inclusion practices such as tailored advisor–advisee pairing, peer mentoring first-year students and a depth and breadth of clubs and activities. One deficient area is related to Hispanic and female students not feeling as included in classes, with peers, and with faculty. Therefore, the DEI-B program must continue to evaluate, evolve, and improve to serve all undergraduate students as this demographic population continues to change.
